# Dzherelo (Immunoxel) as adjunctive therapy to standard antituberculosis treatment in patients with pulmonary tuberculosis: a systematic review and meta-analysis of clinical trials

**DOI:** 10.1186/s13643-021-01698-2

**Published:** 2021-05-26

**Authors:** Marcel Kitenge, Bessie Phiri, Sara M. Pheeha, Modupe Ogunrombi, Peter S. Nyasulu

**Affiliations:** 1 Division of Epidemiology and Biostatistics, Department of Global Health, Faculty of Medicine and Health Sciences, Stellenbosch University, Cape Town, South Africa; 2 Medecins sans Frontieres, Doctors without Borders, Eshowe Project, Eshowe, KwaZulu Natal, South Africa; 3 Clinical Services Ministry of Health, P.O Box 30377, Lilongwe 3, Malawi; 4 National Health Laboratory Services, Dr George Mukhari Hospital/Sefako Makgatho Health Sciences University, Pretoria, South Africa; 5 Department of Pharmacology, Sefako Makgatho Health Sciences University, Pretoria, South Africa; 6 Division of Epidemiology and Biostatistics, School of Public Health, Faculty of Health Sciences, University of the Witwatersrand, Johannesburg, South Africa

**Keywords:** Immunoxel, Dzherelo, Tuberculosis, Antituberculosis treatment (ATT), Adjunctive therapy, Meta-analysis, Clinical trials, Immunotherapy

## Abstract

**Background:**

Dzherelo (Immunoxel) is one of the few approved immunomodulators that has been shown to produce positive treatment outcomes in patients with tuberculosis (TB). The aim of this review was to assess the effectiveness of Immunoxel used as adjunct therapy with conventional anti-TB therapy for the treatment of pulmonary TB.

**Methods:**

Comprehensive search was conducted in different major databases: PubMed (MEDLINE), EMBASE (OVID), Cochrane Central Register of Controlled Trials (CENTRAL), Scopus (Elsevier). We also searched Google Scholar along with trial registries and hand-searched the reference list of identified original research as well as review articles. Conference proceedings of relevant TB and lung disease annual conferences were also screened. Two independent authors extracted outcome data using a standardised extraction form. Relative risk (RR), mean difference (MD) and standardised mean difference (SMD) with a 95% confidence interval (CI) were used as measures of effect. We assessed certainty of evidence using GRADE.

**Results:**

Six clinical trials, which met the criteria for the review, were identified, and these provided data for the review. Overall results from the six trials that compared antituberculosis treatment (ATT) alone versus ATT and Immunoxel, and ATT and placebo versus ATT and Immunoxel showed an increased number of patients becoming sputum-negative in the Immunoxel group (RR 3.19; 95% CI 2.44 to 4.17; 488 participants). There was also reduction in body temperature among patients receiving Immunoxel compared to ATT alone (MD −0.20, 95% CI −0.22 to −0.18, 345 participants). However, there were no differences in body weight changes across all the studies (MD 5.65; 95% CI −0.80 to 12.11; 382 participants).

**Conclusion:**

Current evidence indicates that the use of Immunoxel as an adjunctive treatment in patients with pulmonary tuberculosis has the potential to enhance the efficacy of antituberculosis treatment. However, well-designed, conducted and adequately powered clinical trials are needed to establish the effectiveness of this adjunctive treatment.

**Systematic review registration:**

PROSPERO registration number: CRD42019127823

**Supplementary Information:**

The online version contains supplementary material available at 10.1186/s13643-021-01698-2.

## Background

Tuberculosis (TB) has existed for millennia and remains one of the major public health concerns [1]. In 2019, 1.4 million deaths that are due to TB were recorded including an estimated 10 million new incident cases worldwide [1]. Drug-resistant TB continues to be a threat to TB management despite the fact that, with a timely diagnosis and appropriate treatment, most of the people who contract TB could be cured [1]. The standard treatment regimens for latent TB infection take 3–9 months, and new incident cases of TB require at least 6 months of treatment with multiple drugs [2]. A decline in the success rate of treatment and the increase in multidrug-resistant TB or rifampicin-resistant TB (MDR/RR-TB) indicate the urgent need for better treatment options [1, 2]. One of the interventions that could be employed in addressing these challenges is immunotherapy. This is believed to enhance the efficacy of TB chemotherapy and to potentially shorten the treatment duration [3].

Dzherelo (Immunoxel) is an oral immunomodulatory botanical compound that strengthens the immune system and aids the ability of the host to clear infectious diseases by restoring humoral and cellular immunity and increasing interferon production [3]. Immunoxel is one of the few approved immunomodulators that has been shown to produce positive outcomes in patients with TB. Immunoxel was formulated in 1980 by a Ukrainian scientist Volodymyr Pylypchuk (Ekomed company) and is currently widely available in Ukraine as an oral immunomodulating agent [4]. The Ministry of Health in Ukraine approved the formulation of Immunoxel in 1997 as a dietary herbal supplement following comprehensive laboratory and clinical testing [5]. In 2016, Immunoxel was officially approved as an oral immunomodulator by the Ukrainian Ministry of Health.

Many clinical studies involving patients with or without HIV/TB co-infection were carried out in Ukraine to assess the effectiveness of Immunoxel [5–7]. Approximately 90% of patients included in these studies reported objective improvement in their well-being, as demonstrated by an increase in body weight, improved liver function, and decrease in incidence of opportunistic infections [8]. Moreover, by the 6th month of follow-up, the proportion of cured TB patients by culture and radiology was about 2–4 folds higher than of those who received standard first-line antituberculosis treatment (ATT) alone [5].

Immunoxel can also eliminate *Mycobacterium tuberculosis*, and this is proved by accelerating sputum conversion; improving chest image, especially cavity closure; and improved overall respiratory function as well as clinical features such as reversal of weight loss, correction of the hepatotoxicity caused by TB drugs and increase in the absolute number of CD4 and CD8 T-lymphocytes [6]. In addition, the use of this immunomodulator alongside with ATT has been proven to shorten the duration of treatment as compared to using standard ATT alone [6]. Although several clinical trials have been conducted to date, this evidence has not yet been assessed in a systematic review. We therefore conducted a systematic review of the current existing evidence to assess effectiveness of adjunct Immunoxel with conventional anti-TB therapy for the treatment of pulmonary tuberculosis.

## Materials and methods

The methods of this systematic review and meta-analysis were reported as per the Preferred Reporting Items for Systematic Review and Meta-Analysis Protocols (PRISMA-P) checklist. We registered the protocol for this systematic review on the International Prospective Register of Systematic Reviews (PROSPERO) with a registration number: CRD42019127823.

### Types of studies

We included randomised and non-randomised controlled trials (RCT and non-RCT) that evaluated the adjunct effects of Immunoxel in TB patients allocated to adjunct Immunoxel with standard-of-care anti-TB treatment (standard TB treatment) and standard TB treatment with placebo or standard TB treatment alone.

### Types of participants

We included pulmonary TB patients older than 18 years, irrespective of resistance types.

### Types of interventions

- Intervention group: adjunct Immunoxel with standard-of-care anti-TB treatment (standard TB treatment)
- Comparison group: standard TB treatment with placebo or standard TB treatment alone.

We did not impose any restrictions on study interventions, such as dose, timing of outcomes measurement and duration of treatment.

### Types of outcomes measures

#### Primary outcome

The primary outcome for this review was sputum smear conversion. This is defined as the proportion of patients with sputum that has been converted to negative at a certain point in time after initiation of anti-TB treatment and are therefore no longer infectious.

#### Secondary outcome

##### Safety

We defined safety as the occurrence, after initiation of the study drug treatment, of either:

- An increase in body weight from baseline (*P*<0.05) by the end of study.
- A decrease in levels of alanine transaminase and total bilirubin from baseline (*P*<0.05) by the end of the study.

### Electronic searches

A comprehensive and exhaustive search was performed by MKK, one of the three review authors, with the help of an information specialist to identify relevant studies in the following electronic databases: PubMed (MEDLINE), EMBASE (OVID), Cochrane Central register of controlled trials (CENTRAL), Scopus (Elsevier). We searched Google Scholar and also looked for ongoing RCTs of adjunctive therapies in TB in the following registries:

- US National Institute of Health Ongoing Trials Register (www.clinicaltrials.gov)
- World Health Organization International Clinical Trials Register Platform (WHO ICTRP) (www.who.int/ictrp)
- Pan African Clinical Trials Registry (PACTR) (www.pactr.samrc.ac.za)

Searches were run on 21 May 2020 and were not restricted to date, language, or publication status. Detailed search strategies are presented in Additional file 1.

### Searching other resources

We also hand-searched the reference list of included studies. Conference proceedings of the International Union against Tuberculosis and Lung Disease (IUATLD) World Congress, The European Respiratory Society World Congress Conferences and the American Thoracic Society International Congress were screened in order to retrieve information on any further trials that may not have been included in the electronic database.

### Study selection

Search results were imported into Covidence (Covidence Systematic Review Software, Veritas Health Innovation, Melbourne, Australia), and three review authors (MKK, BP and SM) independently screened the titles and abstracts obtained from the electronic searches, as well as full texts of all potentially eligible studies using a standardised eligibility form with predefined inclusion criteria. Disagreement between the authors who assessed study eligibility was resolved by discussion and consensus.

### Data extraction and management

Two review authors (MKK and BP) independently extracted data from included studies using a standardised data extraction form and performed risk of bias assessment. Extracted information included details of the study, participants, interventions and outcomes. Moreover, we assessed the risk of bias for RCTs using the Cochrane risk of bias for randomised controlled trials as described in the Cochrane Handbook for Systematic Reviews of Interventions [9]. Thus, the assessment of risk of bias took into account the variation in the study designs (RCTs and non-RCTs), as certain criteria were only applicable to RCTs and others were only applicable to non-randomised studies.

Disagreement between authors who extracted the data and assessed the risk of bias was resolved by discussion and consensus. We planned to assess for publication bias using funnel plot, but this was not done due the insufficient number of studies included in this review. Data were entered into the Review Manager 5.4 statistical Software [10].

### Statistical analysis

Dichotomous data were presented and compared using risk ratio while continuous outcomes were presented and compared using mean difference (MD). Furthermore, as studies used different units to measure some of the biochemical parameters, standardised mean difference (SMD) was used to present and compare data, we assumed SMD of 0.2 to represent a small effect, 0.5 a medium effect and 0.8 a large effect accordingly [11]. All measures of effect were reported with their corresponding 95% confidence intervals (CI).

We assessed heterogeneity between trial results by visually inspecting the forest plots for overlapping confidence intervals, followed by the chi-squared test of homogeneity (with significance defined at an alpha of 10%). We then used the I^2^ test to quantify the degree of heterogeneity. We conducted meta-analysis when included studies were similar in terms of interventions, participants, and outcomes. We pooled the results using the Mantel-Haenszel method and fixed model effects. When there was substantial heterogeneity, we used random-effects model. When I^2^ was greater than 50%, we considered it to be substantial heterogeneity and explored the cause of heterogeneity using subgroup analyses. All the analyses were performed using RevMan 5.4.

Finally, GRADE approach [12–14] was used to assess the quality of evidence for the adjunct effect of Immunoxel. We recorded the quality of evidence as high, moderate, low or very low.

## Results

### Study flow chart and description of studies

**Fig. 1:**
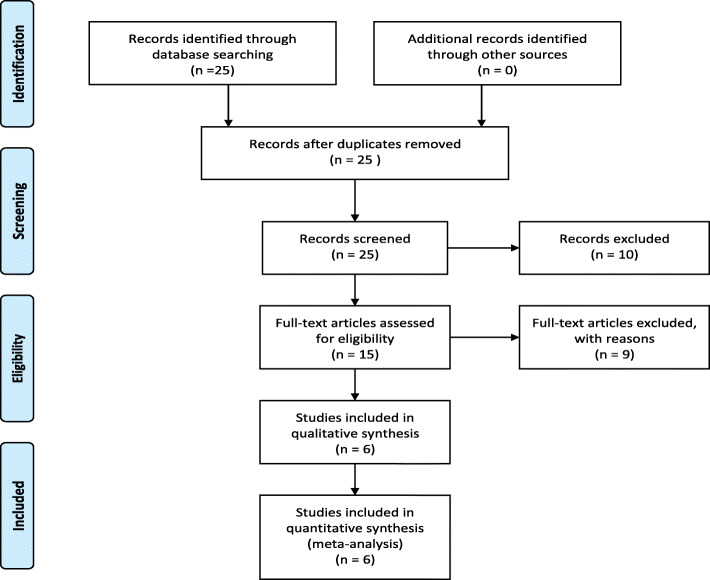
Flow chart for studies in the review

Results of study selection processes are described in a flow diagram (Fig. 1). We identified 25 records through a comprehensive and exhaustive search. Twenty-five titles and abstracts were screened, and 10 articles were deemed to be irrelevant. Following the full-text assessment, independent review and discussion, of the remaining 15 full text articles, we included 6 studies [5–8, 15, 16]. We have provided reasons for excluding irrelevant studies in Table 1.

**Table 1:** Characteristics of excluded studies

Study	Reason for exclusion
Amin [4]	This was a review [4]
Zulkifli et al. [17]	Study was a systematic review [17]
Butov et al. [18]	Study used V-5 Immunitor, an intervention not under this review [18]
Nikolaeva et al. [19]	The study did not report of the outcomes of interest [19]
Nikolaeva et al. [19]	This study [20] was duplicate of study [19]
Nikolaeva et al. [19]	The study did not report any of the outcomes of interest, the study looked particularly at the effect of Dzherelo on immunological and virological responses (T-lymphocyte and viral load among TB/HIV patients) [20].
Prihoda et al. [21]	Not a comparative study, in addition, all the participants received the intervention in combination with some other immunomodulators [21].
Prihoda et al. [22]	The study compared Dzherelo (Immunoxel) with other forms of immunotherapies (Svitanok and Lizorm) [22].
Prihoda et al. [23]	This study [23] was a duplicate of study [21].

### Excluded studies

Zulkifli et al. [17] and Prihoda et al. [21] were excluded because they were not clinical trials. Butov and colleagues [18] were excluded because the intervention used is not under this review. Nikolaeva et al. [19, 20] was excluded because their study did not report any of the study outcomes in this review. Studies [4, 8, 23] were excluded after finding out that they were duplicate studies and therefore were results of a publication bias (studies published in two different journals using different titles). Furthermore, Prihoda et al. [22] was excluded because the study used Immunoxel combined with other forms of immunotherapies. The details for exclusion of studies are provided in Table 1.

### Included studies

We provided detailed information of the included studies and summarise key features (Table 2). All studies were conducted in Ukraine, including one multicentre conducted in Ukraine and Mongolia [5]. Two studies were open-label RCT, one double-blinded placebo RCT, one unblinded RCT and one clinical trial with unspecified methods. Batbold et al. [5] was the most powered of the studies included in this review (269 participants). Zaitzeva et al. enrolled 75 newly diagnosed TB patients [8]. Efremenko et al. randomly allocated 69 participants to one of the four different types of Immunoxel formulations [7]. Furthermore, Zaitzeva et al. matched 66 participants to receive either individualised ATT or ATT with liquid Immunoxel [16]; and Arjanova et al. matched 40 participants to receive either ATT or ATT with Immunoxel [15]. The study population for five trials were in-patients; however, one trial did not clearly specify its study population as to whether they were inpatients or outpatients.

**Table 2:** Characteristics of included studies

Trials	Country	Study design	Number of participants	Intervention	Comparator	Ref
Arjanova et al.	Ukraine	Open-label trial	40 TB/HIV coinfected patients	Immunoxel with ATT	ATT alone	[15]
Arjonova et al.	Ukraine	Open-label trail	40 TB/HIV coinfected patients	Immunoxel with ATT	ATT alone	[6]
Batbold et al.	Ukraine and Mongolia	Double-blinded placebo controlled RCT	269 participants	Immunoxel with ATT	ATT with placebo	[5]
Efremenko et al.	Ukraine	Unblinded RCT	69 patients, 76.8% with TB and 23.2% with TB/HIV co-infection	Various Immunoxel formulations: sugar dragees, sugar-coated pills, gelatin pastilles and dried honey lozenges	Sugar-coated pills without Immunoxel	[7]
Zaitzeva et al.	Ukraine	Non-randomised controlled trial	75 newly PTB patients to assess the adjunct effect of Dzherelo on clinical outcomes and biochemical and blood parameters in patients with cavitary and infiltrating PTB	Immunoxel with ATT	ATT only	[8]
Zaitzeva et al.	Ukraine	Non-randomised controlled trial	66 patients of which 48 had MDR-TB	Immunoxel with ATT	ATT alone	[16]

*ATT* antituberculosis therapy, *RCT* randomised controlled trial

Four studies compared liquid-based formulation, 50 drops of Immunoxel twice daily, with placebo [6, 8, 15, 16]. One study compared four different formulations of Immunoxel given once per day [7], and one study compared unspecified Immunoxel to placebo [5].

### Risk of bias

**Fig. 2:**
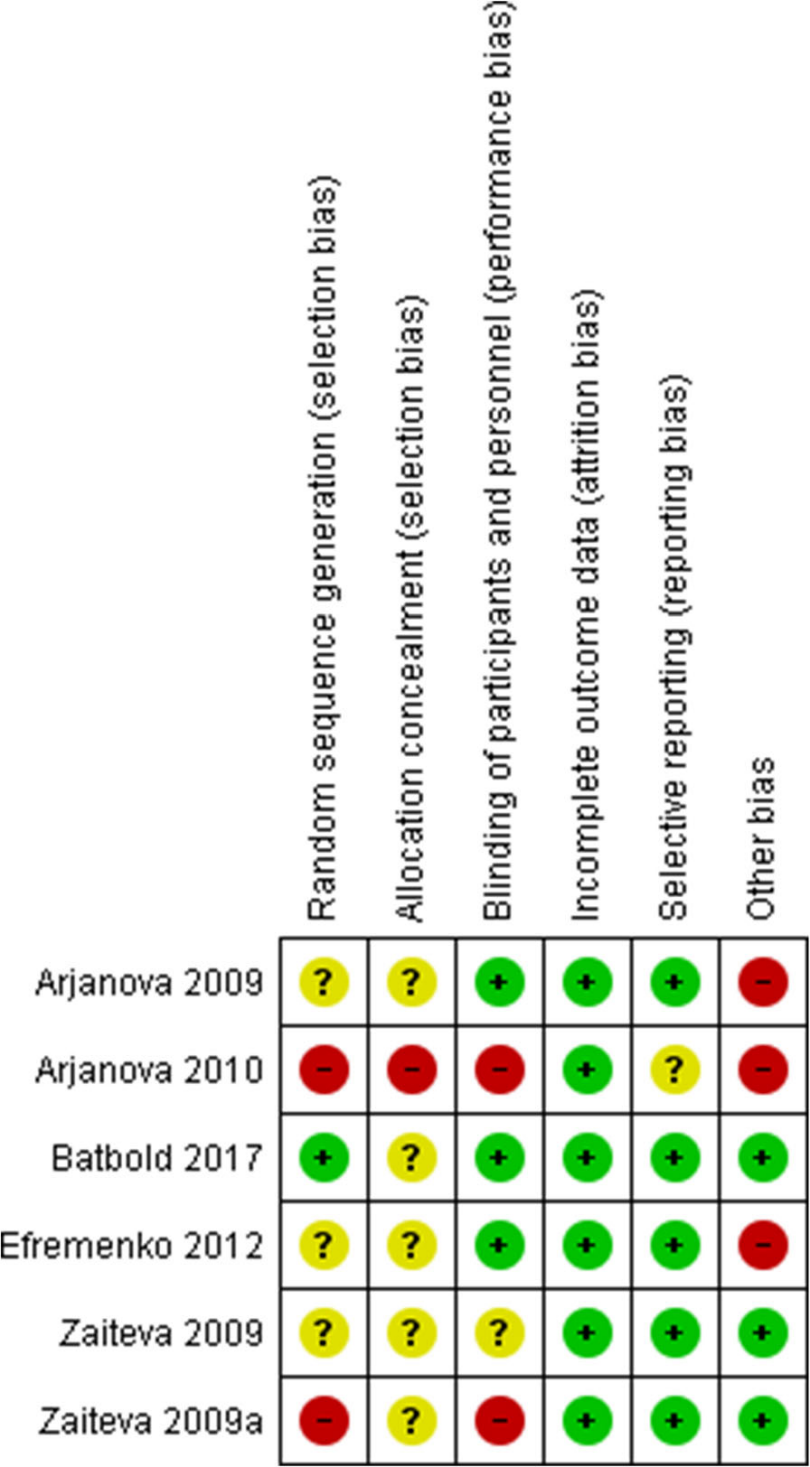
Risk of bias summary: review authors’ judgement about each risk of bias item for each included study

**Fig. 3:**
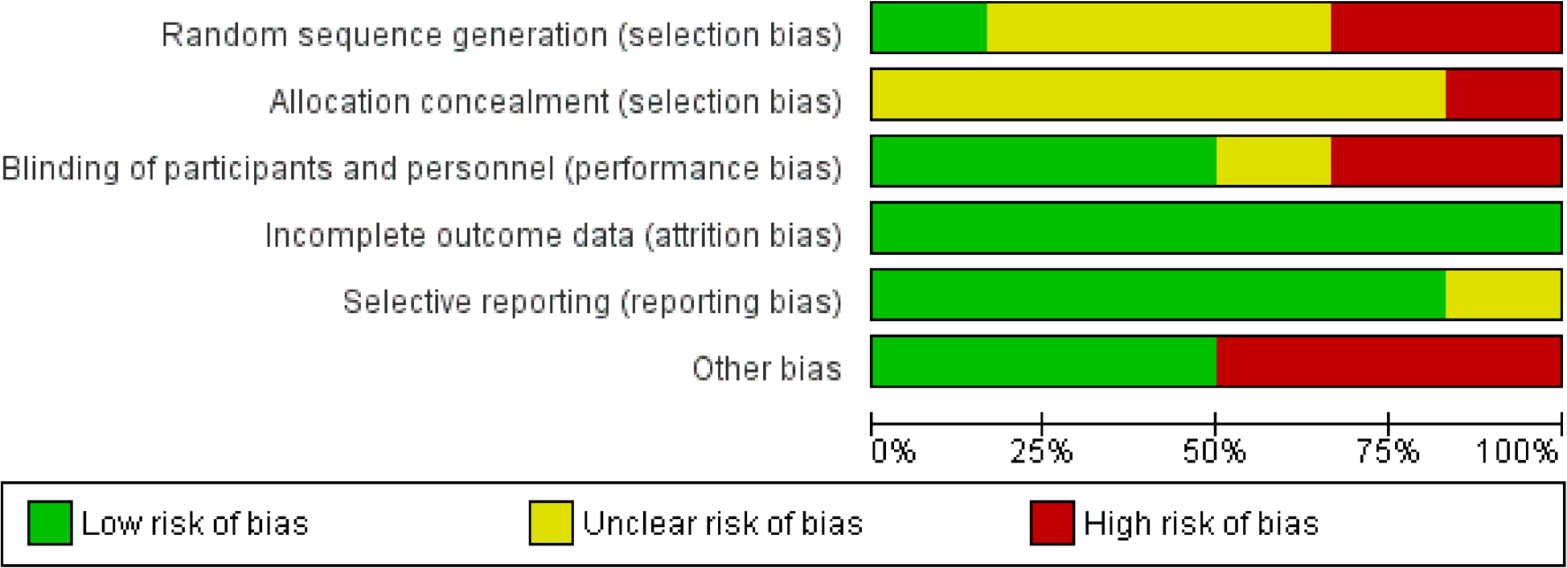
Risk of bias graph: review authors’ judgement about each risk of bias item presented as percentages across all included studies

A graphical representation of the overall risk of bias in the included studies is presented in Figs. 2 and 3. All trials had a higher risk of bias due to unreported, inadequate or unclear methods of random sequence generation and lack of allocation concealment. Three studies [6, 7, 15, 16] did not report how the allocation was generated. Batbold et al. [5] used a computer to generate the allocation while Zaitzeva et al. [8] did not allocate groups randomly. All the studies did not report how the allocations were concealed.

Concerning blinding, Arjanova et al. [15] did not state the exact method used. Batbold et al. [5] reported that neither study personnel nor patients were aware of the intervention. Efremenko et al. [7] reported that only outcome assessors were blinded. Moreover, Zaitzeva et al. did not report the exact method used for blinding [16] and finally Zaitzeva et al. [8] was an open label.

Selective reporting was difficult to assess, considering the fact that none of the studies reported a protocol being available. Nevertheless, primary endpoints were reported as specified in the study objectives. Arjanova et al. [15] as well as Efremenko et al. [7] provided very limited information relative to the methods.

### Effect of interventions on smear conversion

**Fig. 4:**
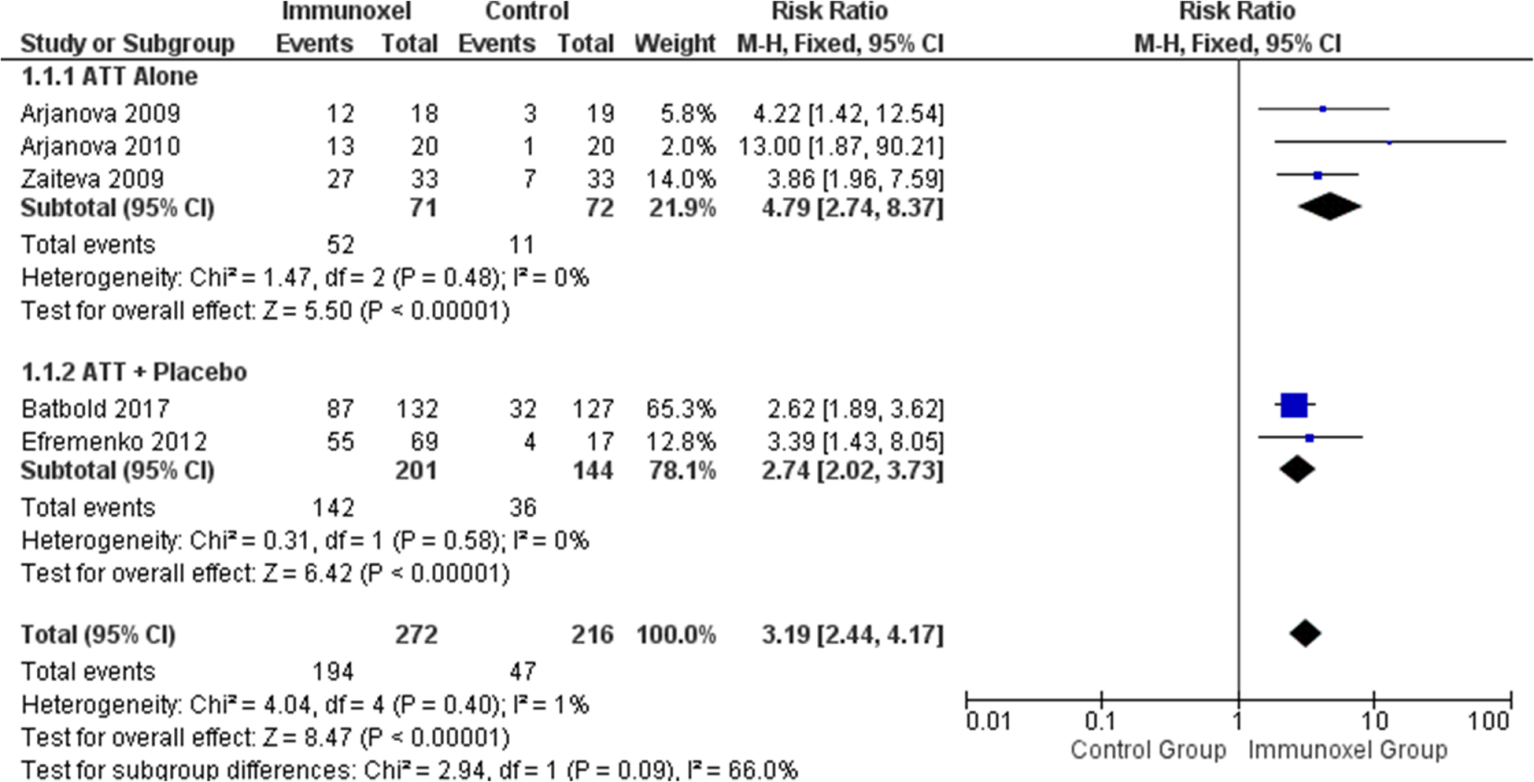
Effect of Immunoxel compared with ATT alone or ATT with placebo on smear microscopy conversion

Five trials including 488 participants contributed to this outcome [5–8, 15]. There was evidence of an increased number of patients becoming sputum-negative in the Immunoxel group (RR 3.19; 95% CI 2.44 to 4.17) (Fig. 4). Heterogeneity was not important among these studies (Chi^2^=4.04, degree of freedom (DF) =4 (P<0.40); I^2^=1%) (Fig. 3). The quality of this evidence was low.

### Effect of interventions on weight change

**Fig. 5:**
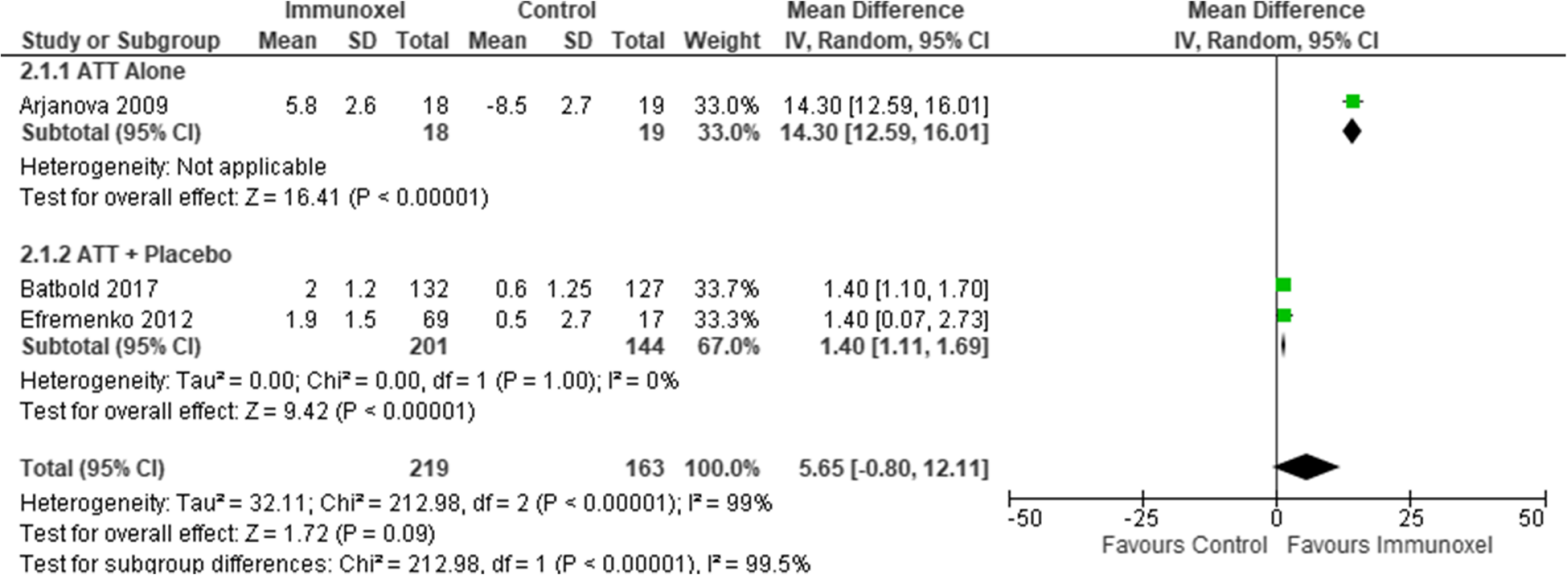
Effect of Immunoxel compared with ATT alone or ATT with placebo on weight change

There were two studies that compared ATT alone with ATT plus Immunoxel [5, 7, 15] and one study that compared ATT alone with ATT plus multiple formulations of Immunoxel [6]. Pooled analysis of data provided by 3 studies with 382 participants showed that there was no evidence of a difference in weight change (MD 5.65, 95% CI −0.80 to 12.11). There was a substantial statistical heterogeneity (Tau^2^= 32.11; Chi^2^=212.98, degree of freedom (DF=2) P<0.00001; I^2^=99%) (Fig. 5) and marked clinical heterogeneity between studies contributing to the outcomes. The quality of this evidence was very low.

We also conducted subgroup analysis for this outcome to investigate heterogeneity. This subgroup analysis evaluated ATT alone versus ATT plus placebo; only one study compared Immunoxel to ATT alone (MD 14.30, 95% CI 12.59 to 19.01) [15]. When analysis of this study was separated from the remaining of the studies, the pooled weight change continues to show an increase among participants who received Immunoxel compared to placebo, and studies included in the analysis were relatively homogenous (MD 1.40, 95% CI 1.11 to 1.69) (Chi^2^=25.62, degree of freedom (DF)=1 (P<0.00001); I^2^=0%). The test for subgroup differences indicated that there is statistically significant subgroup effect (P<0.00001).

### Effect of interventions on level of alanine transaminase

**Fig. 6:**
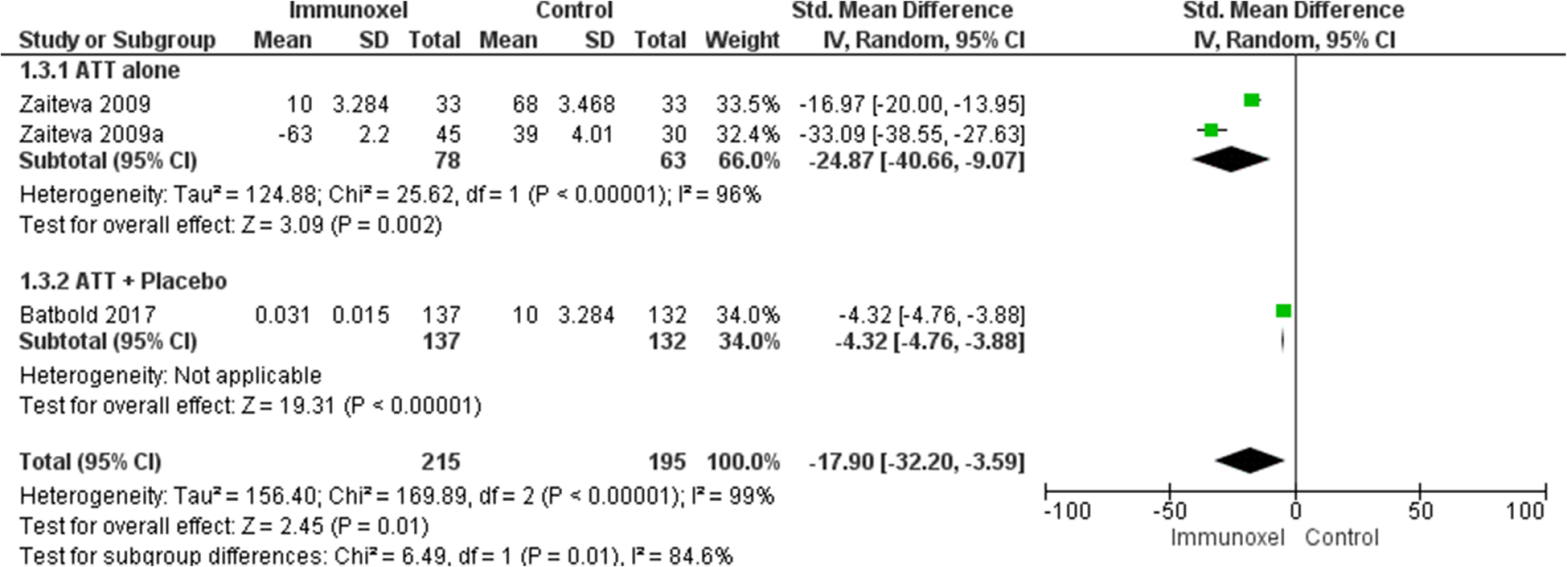
Effect of Immunoxel compared with ATT alone or ATT with placebo on level of alanine transaminase

Three studies including 410 participants contributed to this outcome [5, 8, 16]. There was a large reduction in level of alanine transaminase (ALAT) among participants receiving Immunoxel compared to ATT alone (SMD −17.90, 95% CI −32.20 to −3.59). There was a substantial statistical heterogeneity among these studies (Chi^2^=169.89, degree of freedom (DF) =2, P<0.00001; I^2^=99%) (Fig. 6), and the quality of evidence was very low. A SMD was used to determine the effect of Immunoxel as included studies used different units to measure alanine transaminase. We also conducted subgroup analysis for this outcome to investigate heterogeneity. This subgroup analysis evaluated ATT alone versus ATT plus placebo. There was only one study that compared ATT plus placebo with ATT plus Immunoxel (SMD −4.32, 95% CI −4.76 to −3.88) [5]. When analysis of this study was separated from the remaining of the studies, the pooled ALAT showed larger change among participants who received Immunoxel compared to placebo and considerable heterogeneity persisted in the analysis (SMD −24.87, 95% CI −40.66 to −9.07) (Chi^2^=25.62, degree of freedom (DF) =1 (P<0.00001); I^2^=96%).

### Effect of intervention on total bilirubin

**Fig. 7:**
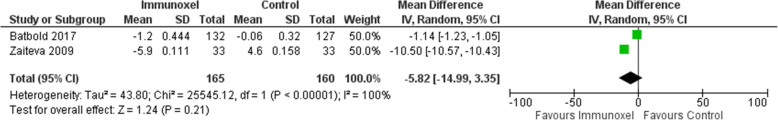
Effect of Immunoxel compared with ATT alone or ATT with placebo on total bilirubin

Two studies evaluated effect of Immunoxel on total bilirubin. The total number of participants in the Immunoxel and control group was 165 and 160, respectively [5, 8]. There was no evidence of bilirubin reduction among participants receiving Immunoxel compared to control (SMD −5.82, 95% CI −14.99 to 3.35). There was a substantial heterogeneity (Tau^2^ =43.80; Chi^2^=25545.12, degree of freedom (DF=1), *P*<0.00001; I^2^=100%) (Fig. 7), and marked clinical heterogeneity between studies contributing to the outcomes and the quality of this evidence was very low.

### Effect of intervention on body temperature

**Fig. 8:**
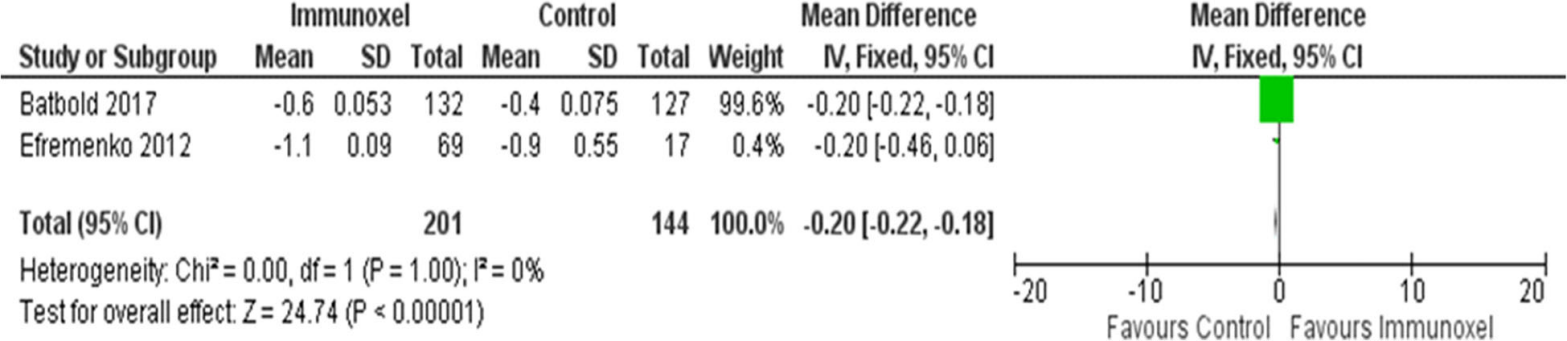
Effect of Immunoxel compared with ATT alone or ATT with placebo on body temperature

Two trials including 345 participants contributed to this outcome [5, 7]. There was evidence of a decreased body temperature among participants in the Immunoxel group (MD −0.20; 95% CI −0.22 to −0.18); homogeneity was not important (Chi^2^=0.00, degree of freedom (DF) =1 (P=1.0); I^2^=0%) (Fig. 8); the quality of this evidence was low.

## Discussion

Our systematic review and meta-analysis aimed to assess the effectiveness of adjunctive Immunoxel therapy for the treatment of pulmonary TB. We found six studies with a total of 563 participants, which addressed five outcomes. There was an overall positive effect of Immunoxel in sputum smear conversion; however, studies contributing to this outcome were small and of poor quality (low quality of evidence). Therefore, poor quality of evidence precludes drawing firm conclusions regarding the effect of the Immunoxel as an adjunctive immunotherapy.

Previous investigations also showed that Immunoxel resulted in a higher rate of clearance of *M. tuberculosis* in sputum cultures than in patients treated with TB drug alone or with placebo [6, 23]. Immunoxel eliminates *Mycobacterium tuberculosis* by accelerating sputum conversion, improving chest image especially cavity closure and also improving overall clinical features and respiratory functions [15, 20]. These positive results are related with better immune response as Immunoxel could regulate both humoral and cellular immune response [20]. Immunoxel plays important roles in the following: (i) higher IL-2 and IFN-ɤ which have a role in accelerated mycobacterium elimination, (ii) suppressed IL-2 which interrupt cellular immune response, (iii) lower TNF-α. The lower TNF-α is usually related with worse result. It was shown that Immunoxel can efficiently reduce pro-inflammatory cytokines [24]; thus, immunotherapies should be aimed at downregulation rather than exacerbating an already intense immune response. However, it is unclear how the downregulation of inflammation correlates with bacterial clearance. The mechanism of action of Immunoxel is unknown, and further studies addressing this question needs to be carried out.

Notably, the remarkable effect of Immunoxel reported across studies is body weight gain, but our review did not find a significant difference in body weight gained. However, an improved liver function and decrease in body temperature were noted. A major limitation of the current review in support of Immunoxel is the small number of the included studies. Small trials can provide firm and definitive answers to questions regarding safety of therapy when outcomes are dichotomous [25]. However, the findings of small trials are misleading due to random error [26]. Moore et al. demonstrated that for the results to be statistically significant and clinically meaningful, 500 participants per comparison group are needed, which can be achieved by conducting a large trial or by pooling results from multiple studies of small size [27]. We understand the rather unusual path that Immunoxel has made on its way to testing in phase 3 studies as literature on preclinical experimental data, toxicity and dose finding is still unknown.

A further challenge in this review is the high degree of heterogeneity observed in the outcomes reported across studies. With an exception of the effect of Immunoxel on smear conversion and body temperature, the remaining outcomes varied widely across the different studies. Another challenge in this review is that the included studies were all conducted in Ukraine, with the exception of one study [5], conducted in both Ukraine and Mongolia, but that also was initiated by the same Ukrainian co-authorship team. This may affect the projections of the inferences from the review to other low- and middle-income settings.

It is unlikely that we missed any relevant RCTs and non-RCT that could have assessed the clinical benefit of Immunoxel used as adjunct with conventional anti-TB therapy for the treatment of pulmonary TB. Apart from the electronic and manual searches, Immunoxel manufacturers were contacted but unfortunately, we could not obtain any information about studies of Immunoxel from the manufacturer. The current review highlights inappropriate reporting of trials by authors, which makes judgement of the quality of the studies challenging. CONSORT guidelines were established for reporting trials; however, it is not always possible in a practical setting to follow all the CONSORT guidelines.

We were unable to formally assess the likelihood of publication bias in this review due to small number of studies included per criterion. However, as publication bias is more likely with small trials, this could be an alternative explanation of the positive findings seen in the studies we identified [28].

### Quality of evidence

We used the GRADE approach to assess the certainty of evidence as shown in the summary of findings table in Additional file 2. The overall quality of evidence in this review on the use of Immunoxel as adjunctive treatment in patient with pulmonary tuberculosis is low due to high risk of bias. Additionally, we also observed substantial heterogeneity in included studies. Based on the above quality of evidence, the implication is that there is need for further research particularly involving RCT design in order to enhance our quality of evidence.

### Implications for practice and future research

The current evidence suggests the use of Immunoxel as adjunctive treatment in patients with pulmonary tuberculosis. However, the paucity of data encountered suggests that there is room for more rigorous and carefully designed clinical trials to confirm these findings. Since RCT are the gold standard for testing the effect of new treatment, it would be useful to see adequately powered trials to establish the value of Immunoxel as adjunctive treatment in patients with pulmonary tuberculosis and increase the certainty of the current evidence base, preferably in placebo-controlled settings. Careful attention should be given to the outcomes to be assessed in future trials to ensure that selected outcomes are important to patients and are measured in a standardised manner and longer duration.

## Conclusion

The findings of this systematic review indicate that the use Immunoxel as adjunctive treatment in patients with pulmonary tuberculosis has the potential to enhance the efficacy of the antituberculosis treatment. However, in order to draw a firm conclusion, methodologically rigorous and well-reported trials are required to confirm these findings. The results of this systematic review also lay an important foundation on which further studies could be built on.

## Supplementary Information

**Additional file 1.** Search strategies.

**Additional file 2.** Summary of findings table.

**Additional file 3.** Data Extraction form.

## Data Availability

All data generated or analysed during this study are included in this published article and its additional files.

## Abbreviations

ALAT: Alanine transaminase
ATT: Antituberculosis treatment
CONSORT: Consolidated Standard of Reporting Trials
DF: Degree of freedom
GRADE: Grading of Recommendations, Assessment, Development and Evaluations
HIV: Human immunodeficiency virus
IUATLD: International Union Against Tuberculosis and Lung Disease
MD: Mean difference
MDR-TB: Multidrug-resistant tuberculosis
PRISMA-P: Preferred Reporting Items for Systematic Review and Meta-Analysis Protocols
PROSPERO: Prospective Register of Systematic Reviews
RCT: Randomised controlled trials
RR: Relative risk
SMD: Standardised mean difference
TB: Tuberculosis

## References

[1] World Health Organization. Global tuberculosis report 2020. Geneva: World Health Organization; 2020. Licence: CC BY-NC-SA 3.0 IGO. Available at https://www.who.int/publications/i/item/9789240013131.

[2] Zumla A, Chakaya J, Centis R, Ambrosio LD, Mwaba P, Bates M (2015). Tuberculosis treatment and management — an update on treatment regimens, trials, new drugs, and adjunct therapies. Lancet Respir Med..

[3] Abate G, Hoft D (2016). Immunotherapy for tuberculosis: future prospects. Immunotargets Ther..

[4] Zulkifli Amin SPH. Promising herbals as adjunctive to standard antituberculosis therapy. Indian J Public Health. 2020;11(1):1–8.

[5] Batbold U, Butov DO, Kutsyna GA, Damdinpurev N, Grinishina EA, Mijiddorj O, Kovolev ME, Baasanjav K, Butova TS, Sandagdorj M, Batbold O, Tseveendorj A, Chunt E, Zaitzeva SI, Stepanenko HL, Makeeva NI, Mospan IV, Pylypchuk VS, Rowe JL, Nyasulu P, Jirathitikal V, Bain AI, Tarakanovskaya MG, Bourinbaiar AS (2017). Double-blind, placebo-controlled, 1:1 randomized phase III clinical trial of Immunoxel honey lozenges as an adjunct immunotherapy in 269 patients with pulmonary tuberculosis. Immunotherapy..

[6] Arjanova OV, Prihoda ND, Yurchenko LV (2010). Impact of adjunct immunotherapy with multi-herbal supplement Dzherelo (Immunoxel) on treatment outcomes in end-stage TB/HIV patients. J Antivirals..

[7] Efremenko YV, Arjanova OV, Prihoda ND, Yurchenko LV, Sokolenko NI, Mospan IV, Pylypchuk VS, Rowe J, Jirathitikal V, Bourinbaiar AS, Kutsyna GA (2012). Clinical validation of sublingual formulations of Immunoxel (Dzherelo) as an adjuvant immunotherapy in treatment of TB patients. Immunotherapy..

[8] Zaitzeva SI, Matveeva SL, Gerasimova TG, Pashkov YN, Butov DA, Pylypchuk VS, Frolov VM, Kutsyna GA (2009). Treatment of cavitary and infiltrating pulmonary tuberculosis with and without the immunomodulator Dzherelo. Clin Microbiol Infect..

[9] Green S, Higgins JPT, Alderson P, Clarke M, Mulrow CD, Oxman AD, Higgins JPT, Green S (2008). Chapter 1: Introduction. Cochrane Handbook for Systematic Reviews of Interventions.

[10] RevMan (2014). The Nordic Cochrane Centre, the Cochrane collaboration. Review Manager (RevMan). Version 5.3 Copenhagen.

[11] Cohen J (1988). Statistical power analysis for the behavioural sciences.

[12] Dijkers M. Introducing GRADE: a systematic approach to rating evidence in systematic reviews and to guideline development. KT Update. Austin, TX: SEDL, Center on Knowledge Translation for Disability and Rehabilitation Research. 2013;(1)5.

[13] Atkins D, Best D, Briss PA, Eccles M, Falck-Ytter Y, Flottorp S, Guyatt GH, Harbour RT, Haugh MC, Henry D, Hill S, Jaeschke R, Leng G, Liberati A, Magrini N, Mason J, Middleton P, Mrukowicz J, O'Connell D, Oxman AD, Phillips B, Schünemann HJ, Edejer T, Varonen H, Vist GE, Williams JW Jr, Zaza S, GRADE Working Group (2004). Grading quality of evidence and strength of recommendations. BMJ..

[14] Guyatt G, Oxman AD, Akl EA, Kunz R, Vist G, Brozek J, Norris S, Falck-Ytter Y, Glasziou P, DeBeer H, Jaeschke R, Rind D, Meerpohl J, Dahm P, Schünemann HJ (2011). GRADE guidelines: 1. Introduction - GRADE evidence profiles and summary of findings tables. J Clin Epidemiol..

[15] Arjanova OV, Prihoda ND, Yurchenko LV, Sokolenko NI, Vihrova LA, Pylypchuk VS, Frolov VM, Kutsyna GA (2009). Enhancement of efficacy of tuberculosis drugs with Immunoxel (Dzherelo^TM^) in HIV-infected patients with active pulmonary tuberculosis. Immunotherapy..

[16] Zaitzeva SI, Matveeva SL, Gerasimova TG, Pashkov YN, Butov DA, Pylypchuk VS, Frolov VM, Kutsyna GA (2009). Efficacy and safety of phytoconcentrate Dzherelo (Immunoxel) in treatment of patients with multi-drug resistant TB (MDR-TB) in comparison to standard chemotherapy. Res J Med Sci..

[17] Zulkifli A, Purnama H.S. Promising herbals as adjunctive to standard antituberculosis therapy. Indian J Public Heal Res Dev. 2017; 8 (2):220-225

[18] Butov DA, Efremenko YV, Prihoda ND, Yurchenko LI, Sokolenko NI, Arjanova OV, et al. Adjunct immune therapy of first-diagnosed TB, relapsed TB, treatment-failed TB, multidrug-resistant TB and TB/HIV. Futur Med. 2012:687–95. 10.2217/imt.12.59 22853755

[19] Nikolaeva LG, Maystat TV, Masyuk LA, Pylypchuk VS, Volyanskii YL, Kutsyna GA. Changes in CD4+ T-cells and HIV RNA resulting from combination of anti-TB therapy with Dzherelo in TB/HIV dually infected patients. Drug Des Devel Ther. 2009;2:87–93. PMC2761183 19920896

[20] Nikolaeva LG, Maystat TV, Pylypchuk VS, Volyanskii YL, Masyuk LA, Kutsyna GA (2009). Effect of oral immunomodulator Dzherelo in TB/HIV co-infected patients receiving anti-tuberculosis therapy under DOTS. Int Immunopharmacol..

[21] Prihoda ND, Arjanova OV, Yurchenko LV, Sokolenko NI, Vihrova LA, Pylypchuk VS (2007). Open label trial of adjuvant immunotherapy with Dzherelo, Svitanok and Lizorm, in MDR-TB, XDR-TB and TB/HIV co-infected patients receiving anti-tuberculosis therapy under DOT. J Med Plants Res..

[22] Prihoda ND, Arjanova OV, Yurechenko LV, Sokolenko NI, Vihrova LA (2009). Adjuvant immunotherapy of extensively drug resistant tuberculosis (XDR-TB) in Ukraine. Curr Res Tuberc..

[23] Prihoda ND, Arjanova OV, Yurchenko LV, Sokolenko NI, Vihrova LA, Pylypchuk VS (2008). Adjunct immunotherapy of tuberculosis in drug-resistant TB and TB/HIV co-infected patients. Int J Biomed Pharm Sci..

[24] Nikolaeva LG, Maystat TV, Pylypchuk VS, Volyanskii YL, Frolov VM, Kutsyna GA (2008). Cytokine profiles of HIV patients with pulmonary tuberculosis resulting from adjunct immunotherapy with herbal phytoconcentrates Dzherelo and Anemin. Cytokine..

[25] Sackett DL, Cook DJ (1993). Can we learn anything from small trials?. Ann NY Acad Sci..

[26] Guyatt GH, Mills EJ, Elbourne D (2008). In the era of systematic reviews, does the size of an individual trial still matter?. PLoS Med..

[27] Moore RA, Gavaghan D, Tramèr MR, Collins SL, McQuay HJ (1998). Size is everything - large amounts of information are needed to overcome random effects in estimating direction and magnitude of treatment effects. Pain..

[28] Faber J, Fonseca LM (2014). How sample size influences research outcomes. Dental Press J Orthod..

