# Supplementary material for: Dzherelo (Immunoxel) as adjunctive therapy to standard antituberculosis treatment in patients with pulmonary tuberculosis: a systematic review and meta-analysis of clinical trials
Source: Syst Rev. 2021 May 26;10:157. doi: 10.1186/s13643-021-01698-2 (PMC8157410; doi:10.1186/s13643-021-01698-2)
Supplement: Supplementary file 1 — Additional file 1. Search strategies. [file 13643_2021_1698_MOESM1_ESM.docx]

# Additional file 1: Search strategies

1. **Pubmed**

Search strategy

-------------------------------------------------------------------------------

("antitubercular agents"[MeSH Terms] OR ((("tuberculosis"[Title/Abstract] OR "TB"[Title/Abstract]) OR "tuberculosis"[MeSH Terms]) OR ("MDR-TB"[Title/Abstract] OR "XDR-TB"[Title/Abstract]))) AND (("dzherelo"[Supplementary Concept] OR "dzherelo"[All Fields]) OR "Immunoxel"[All Fields])

1. **Database: Embase 1947-Present, updated daily**

Search Strategy

--------------------------------------------------------------------------------

1 tuberculosis.mp. or tuberculosis/ (311371)

2 (MDR-TB or XDR-TB).mp. [mp=title, abstract, heading word, drug trade name, original title, device manufacturer, drug manufacturer, device trade name, keyword, floating subheading word, candidate term word] (6110)

3 multidrug-resistant tuberculosis.mp. or multidrug resistant tuberculosis/ (9170)

4 extensively drug resistant tuberculosis/ (1989)

5 TB.mp. (94930)

6 1 or 2 or 3 or 4 or 5 (343629)

7 immunoxel.mp. (14)

8 dzherelo.mp. (24)

9 7 or 8 (26)

10 6 and 9 (15)

1. **Scopus**

Search Strategy

--------------------------------------------------------------------------------

( TITLE-ABS-KEY ( tuberculosis OR tb OR mycobacterium OR tuberculous OR mdr-tb OR xdr-tb ) AND TITLE-ABS-KEY ( dzherelo OR immunoxel ) )

1. **Cochrane Central Register of Controlled Trials**

Issue 5 of 12, May 2020

Search Strategy

--------------------------------------------------------------------------------

#45 tuberculosis or tuberculous or TB (10864)

#46 MDR-TB or XDR-TB (237)

#47 MeSH descriptor: [Tuberculosis] explode all trees (597)

#48 #45 or #46 or #47 (10864)

#49 immunoxel or dzherelo (8)

#50 #48 and #49 (7)

1. **Google Scholar**: dzherelo, immunoxel
2. **Clinicaltrials.gov**: dzherelo, immunoxel
3. **WHO ICTRP**: dzherelo, immunoxel
