# Supplementary material for: Dzherelo (Immunoxel) as adjunctive therapy to standard antituberculosis treatment in patients with pulmonary tuberculosis: a systematic review and meta-analysis of clinical trials
Source: Syst Rev. 2021 May 26;10:157. doi: 10.1186/s13643-021-01698-2 (PMC8157410; doi:10.1186/s13643-021-01698-2)
Supplement: Supplementary file 2 — Additional file 2. Summary of findings table. [file 13643_2021_1698_MOESM2_ESM.docx]

# Additional file 2. Summary of findings table

| Summary of findings table of Immunoxel with anti-tuberculosis treatment compared to ATT alone or ATT with placebo as adjunctive therapy in patients with pulmonary Tuberculosis | | | | | | |
| --- | --- | --- | --- | --- | --- | --- |
| **ATT with Immunoxel compared to ATT alone or ATT with placebo for ss adjunctive therapy in patient with Pulmonary Tuberculosis** | | | | | | |
| **Patient or population**: Patient with pulmonary Tuberculosis  **Setting**: None  **Intervention**: ATT with Immunoxel  **Comparison**: ATT alone or ATT with placebo | | | | | | |
| Outcomes | **Anticipated absolute effects^*^** (95% CI) | | Relative effect (95% CI) | № of participants  (studies) | Certainty of the evidence (GRADE) |  |
|  | **Risk with ATT alone or ATT with placebo** | **Risk with ATT with Immunoxel** |  |  |  |  |
| Sputum smear conversion | 235 per 1,000 | **702 per 1,000** (535 to 920) | **RR 2.99** (2.28 to 3.92) | 448 (5 RCTs) | ⨁⨁◯◯ LOW ^a^ |  |
| Weight Change | The mean weight Change was **5.65** Kg | MD **5.65 Kg higher** (0.8 lower to 12.11 higher) | - | 382 (3 RCTs) | ⨁◯◯◯ VERY LOW ^b^ |  |
| Level of alanine transaminase | - | SMD **33.98 SD lower** (81.05 lower to 13.09 higher) | - | 325 (2 RCTs) | ⨁◯◯◯ VERY LOW ^c^ |  |
| Total bilirubin | The mean total bilirubin was **5.82** mg/dL | MD **5.82 mg/dL lower** (14.99 lower to 3.35 higher) | - | 325 (2 RCTs) | ⨁◯◯◯ VERY LOW ^c^ |  |
| Body temperature | The mean body temperature was **0.2** Degree Celsius | MD **0.2 Degree Celsius lower** (0.22 lower to 0.18 lower) | - | 345 (2 RCTs) | ⨁⨁◯◯ LOW ^d^ |  |
| ***The risk in the intervention group** (and its 95% confidence interval) is based on the assumed risk in the comparison group and the **relative effect** of the intervention (and its 95% CI).  **CI:** Confidence interval; **RR:** Risk ratio; **MD:** Mean difference; **SMD:** Standardised mean difference | | | | | | |
| **GRADE Working Group grades of evidence** **High certainty:** We are very confident that the true effect lies close to that of the estimate of the effect **Moderate certainty:** We are moderately confident in the effect estimate: The true effect is likely to be close to the estimate of the effect, but there is a possibility that it is substantially different **Low certainty:** Our confidence in the effect estimate is limited: The true effect may be substantially different from the estimate of the effect **Very low certainty:** We have very little confidence in the effect estimate: The true effect is likely to be substantially different from the estimate of effect | | | | | | |

E**xplanations**

a. There is serious concern regarding risk bias, all the included studies had a higher risk of bias due to unreported, inadequate or unclear methods of random sequence generation and lack of allocation concealment. We also noted imprecision due to limited number of participants and studies included

b. Risk of bias due to unreported, inadequate, or unclear methods of random sequence generation and lack of allocation concealment. Inconsistency was also a major concern as there were considerable heterogeneity (heterogeneity: Tau2=32.11; Chi^2^=212.98, df=2 (P=0.00001); I^2^=99%).

c. There is serious concern regarding risk bias, most included studies had a higher risk of bias arisen from the randomization process. In addition, inconsistency and imprecision were major concerns, imprecision due to limited number of included studies and there was substantial statistical heterogeneity and marked clinical heterogeneity.

d. Imprecision due to limited number of participants and studies included. High risk of bias arisen from the randomization process.
