# Supplementary material for: Dzherelo (Immunoxel) as adjunctive therapy to standard antituberculosis treatment in patients with pulmonary tuberculosis: a systematic review and meta-analysis of clinical trials
Source: Syst Rev. 2021 May 26;10:157. doi: 10.1186/s13643-021-01698-2 (PMC8157410; doi:10.1186/s13643-021-01698-2)
Supplement: Supplementary file 3 — Additional file 3. Data Extraction form. [file 13643_2021_1698_MOESM3_ESM.docx]

# Additional file 3. Data Extraction form

| Author year | Country | Randomised (N) | Study design (parallel group or cross over) | Settings (inpatients or outpatients) | TB types | Immunoxel regimen (pills or liquid based) | Comparator (ATT alone or ATT+ Placebo) | ATT Regimen (First or second line) | Comorbidity |
| --- | --- | --- | --- | --- | --- | --- | --- | --- | --- |
|  |  |  |  |  |  |  |  |  |  |

| **Values** | **Author** | |
| --- | --- | --- |
|  | **Intervention** | **Comparator** |
| Number of patients smear converted (n/N) |  |  |
| Time to smear conversion (weeks) |  |  |
| BMI change (kg/m2) |  |  |
| % of patients with healed Cavitary lesions |  |  |
| Adverse events |  |  |
| Level of alanine transaminase (ALT) (U/L) (Mean ALT change) |  |  |
| Level of aspartate transaminase (AST) (Mean AST change) |  |  |
| Bilirubin (µM/L) ( Mean Bilirubin change ) |  |  |
| Body temperature (mean±SD) degree Celsius |  |  |
| Lymphocytes (mean) |  |  |
| leucocytes (mean) |  |  |
| Erythrocytes SR |  |  |
| Haemoglobin |  |  |
| CD4 cell count |  |  |
| CD8 |  |  |
| Comment |  | |
